# Twelfth Report to the Legislature of Massachusetts, Relating to the Registry and Returns of Births, Marriages, and Deaths, in the Commonwealth, for the Year Ending December 31st, 1853

**Published:** 1855-05

**Authors:** Ephraim M. Wright

**Affiliations:** Secretary of the Commonwealth


### BUFFALO MEDICAL JOURNAL

AND

### MONTHLY REVIEW.

**VOL. 10.** MAY, 1855. **NO. 12.**

### ORIGINAL COMMUNICATIONS.

ART. I.—*Twelfth Report to the Legislature of Massachusetts, relating to the Registry and Returns of Births, Marriages, and Deaths, in the Commonwealth, for the year ending December* 31*st,* 1853. By EPHRAIM M. WRIGHT, Secretary of the Commonwealth.

The above is the comprehensive title of a volume of 176 pages just issued by the State of Massachusetts, filled with figures and tables exhibiting the statistics of the subjects treated of in the Report, and illustrative of the deductions to be drawn therefrom. The Returns are for the year 1853, being compiled and arranged during the subsequent year, and submitted to the Legislature on the 30th of Dec., 1854. Ample time has consequently been allowed for the careful preparation of the Report, and a valuable one has been the result.

"The Abstracts have been compiled and prepared from the original returns forwarded to this office, under the active superintendence of NATHANIEL B. SHURTLEFF, M. D., of Boston, who had charge of the Registration Report of the last year, and to whom has been intrusted the preparation of the concluding portion of the Report, embracing the Results and Practical Observations.

"The present Report contains the accumulated results of more than twelve years, and should, therefore, serve as a fair criterion in America wherefrom to deduce facts relative to vital and mortuary statistics as existing in this country."

In view of the failure of the Registration Laws of this State passed a few years since, and of the indifference manifested by the public, professional as well as nonprofessional, to the gathering of such statistics, it is gratifying to have the evidence that this apathy is not universal, but that there is a State which appreciates the value of the facts to be learned from such labors, and has both the spirit to propose, and the vigor to execute the necessary enactments required for the collection of such materials as are embraced in this Report.

The amount of labor bestowed upon this volume, and the character and value of the statistics, may be inferred by the few extracts we propose to make, but a full appreciation of them can be obtained only by a perusal of the Report itself.

Under the head of *Population*, several pages are occupied with the discussion of the number of the inhabitants of the State, which is given by towns and counties, the ratio of increase, their nativity, &c. The United States Census of 1850, is made the basis of these presentations, and furnishes also the ground work for all of the calculations of per centage made in the Report.

"In 1850, the classification of the inhabitants was:

| | Males. | Females. | Total. |
|---|---|---|---|
| White, - - - | 484,093 | 501,357 | 985,450 |
| Free colored, - - | 4,424 | 4,640 | 9,064 |
| Total, - | 488,517 | 505,997 | 994,514 |

"With a population of 994,514 souls, Massachusetts, in 1850, contained

192,675 families,
152,835 dwellings,
15.37 dwellings to 100 persons,
19.37 families to 100 persons,
126.06 families to 100 dwellings.

"Still further reduced, this gives about five persons to a family, between six and seven persons to each dwelling, and requires very nearly four dwellings for five families."

In June, 1850, the proportion born by the sexes to each other, was:

| | | |
|---|---|---|
| (Whites,) | Males, - - - - - - - | 484,093 |
| | Females, - - - - - - | 501,357 |
| (Colored,) | Males, - - - - - - - | 4,424 |
| | Females, - - - - - - | 4,640 |
| | Total, - - - - - - | 994,514 |

"Between the ages of fifteen and thirty, and from fifty upward, the females preponderated among the white population. Among the colored, the number of females exceeded that of males, at all ages, except from twenty to forty.

"The largest number of persons living at the time of taking the census, were of an age between twenty and thirty, and the next greatest number belonged to the next following period of ten years. This holds equally true among the whites and the free colored.

"In 1850, the number of American born citizens in Massachusetts, was 695,236; that of foreigners, 160,909; and the nativity of 3,539 was unknown. Since the material was collected for the census, the immigration of foreigners has been much more extensive."

This subject is farther elaborated by tables of the nativities of the Bostonians, the quota Massachusetts has furnished from her native born sons to people other states, &c.

*Births.*—The whole number of births in the State during the year 1853, as registered by the Town Clerks, was 30,920, being an increase of 1,118 over the number reported the previous year.

| | | | |
|---|---|---|---|
| Of these there were males, - - - | 15,798 | or 51.09 | per cent. |
| " " " " females, - - - | 14,965 | or 48.45 | " " |
| " " sex could not be ascertained, | 157 | or .46 | " " |

The parentage of these is thus represented:

| | |
|---|---|
| The number of those both of whose parents were of foreign birth, was - - - - - - - - - - - - - - - - - - | 11,753 |
| Of those who had American fathers and foreign mothers, there were - - - - - - - - - - - - - - - - - - | 683 |
| Of those of foreign fathers and American mothers, the number was - - - - - - - - - - - - - - - - - - - | 811 |
| Making of those of whom one or both parents were of foreign nativity, - - - - - - - - - - - - - - - - - | 13,247 |

Or 42.85 per cent. of all the children born within the State during the year.

Only 16,040, or 51.88 per cent. were of American parents; the remainder 1,633 or 5.27 per cent. being of undetermined nativity.

These figures represent an increase in the ratio of the births of children of foreign parentage.

The largest number of births occurred in the month of August; 2,887 children being born, of which 1,511 were boys, and 1,362 girls, and 14 the sex not reported. The least fruitful months were January, February, and April.

There were 520 children the result of twin cases, 256 being males, 259 females, and 5 of unknown sex.

During the year there were five cases of triplets:

| | | |
|---|---|---|
| Two | comprising | two males and one female. |
| One | " | all males. |
| One | " | all females. |
| One | " | one male and two females. |

One birth we observe returned as a *hermaphrodite* among the "unknown" sex.

There were 568 still-born children registered during the year. Of these 278 were males, 178 females, and 112 are classed among the unascertained. The reporter stops sufficiently long to complain of the negligence in obtaining and returning the particulars of the sex in these cases. A similar neglect is committed by those who make the returns for our own city; these, omissions, however, affect our local mortuary records only, there being no general registration.

It is stated that the excess of male births over females in the State, for five years, 1849 to 1853, is 4,794. During the last year there has been a slight increase of per centage in favor of the females; yet the males still hold the advantage in the number of births as they have done yearly for the last five years.

These facts thus exhibited are believed to be the uniform results of the observations upon this point throughout the United States, if not throughout the whole civilized world, of the excess of male over female births.

*Marriages.*—During the year 1853, the whole number of marriages solemnized throughout the State, was 12,828, being an increase of 1,250 over the previous year. Two towns are reported as having been without a wedding throughout the year. The following curious facts are deduced from the examination of the records kept for the last ten years:

"It appears that November continues to be the favorite month for the celebration of marriages, more than thirteen per cent. of all the marriages having occurred in that month. This holds true not only for the aggregate of the ten years, but also fer each of the ten years severally. October, May, September, January, and April, follow next in order, closing with March. During the past year January followed next to October. The time of the religious holydays, especially the seasons of Thanksgiving and Fast Days, has generally been most favorable for matrimony."

The most common age in that State, as shown by the tables, is for a first marriage of both parties, from 20 to 25 years, for both sexes. There were 178 recorded were both parties were under twenty years of age.

A good many other curious and interesting details under this head, are omitted for want of room.

*Deaths.*—A multitude of tables, prepared with the most pains-taking labor, exhibit the mortuary statistics gathered throughout the State. But a very faint idea of the minutiæ of details of these figures can be here given.

The whole number of deaths reported for the year was 20,301, being 1,819 more than for the year 1852, but is 122 less than the number for 1849. The number of males dying in 1853 was 9,942, and females 10,201, and 149 whose sex was not obtained.

"The number of deceased persons whose ages were ascertained, amounted to 19,951, making an aggregate of 536,027 years for the whole number, or an average of 26.87 years to each individual. This is a loss of nearly a year when compared with the last year, which showed an average of 27.78 years for each person."

The number of persons dying under five years of age during this year was 7,912, being 38.97 per cent. of all the deaths during the year. After having passed the age of youth and entered into manhood, the most fatal period was between the ages of twenty and thirty.

"On an examination of the tables accompanying the report, it will be perceived that a majority of the children who die early are males, but after the adult period the deaths of females are in excess. On this point the tables of registration for all countries generally agree.

"The greatest mortality throughout the State was in August and September, as was indicated, also, by the returns of the year 1852. These usually prove the most sickly months of the year in Massachusetts, as the tables of former reports conclusively show. In 1853, the deaths in August were 2,484, and in September 2,183. The other months rank in respect to mortality in the following order: July, October, March, December, April, May, January, February, November, and June."

There had been no unusual prevalence of epidemics in the State during the year.

A carefu. and elaborate tabulated exhibition of the causes of death is given too extensive to be attempted here. The causes of death of 20,869 individuals is given and classified in a manner to exhibit at once the males and females, and those of unknown sex, by months, and at various periods of life, arranged in decades.

After deducting the number of 568 still-born, there remain 20,301 who are registered as having died from known causes. But no causes have been assigned to 740 deaths.

"The following table has been carefully prepared in order to exhibit in a clear and comprehensive view the deaths that have occurred during the year 1853, together with those that have been reported the previous eleven years and eight months, as they are found in this and the previous Registration Reports:

| CAUSES OF DEATH. | WHOLE NO. OF DEATHS. | | PER CENTAGE OF DEATHS | |
|---|---|---|---|---|
| | One Year. 1853. | Eleven years and eight months ending Dec. 31, 1852. | One Year. 1853. | Eleven years and eight months ending Dec. 31, 1852. |
| All Causes, | 20,301 | 148,024 | | |
| Specified Causes, | 19,561 | 138,451 | 100. | 100. |
| I. Zymotic Diseases, | 5,446 | 40,681 | 27.84 | 29.37 |
| *Sporadic Diseases of* | | | | |
| II. Uncertain Seat, | 2,409 | 16,862 | 12.32 | 12.17 |
| III. Nervous Organs, | 2,008 | 13,427 | 10.27 | 9.69 |
| IV. Respirative Organs, | 5,783 | 38,713 | 29.56 | 27.94 |
| V. Circulative Organs, | 475 | 2,883 | 2.43 | 2.08 |
| VI. Digestive Organs, | 1,191 | 8,981 | 6.09 | 6.55 |
| VII. Urinative Organs, | 88 | 608 | .45 | .44 |
| VIII. Generative Organs, | 222 | 1,575 | 1.13 | 1.14 |
| IX. Locomotive Organs, | 113 | 752 | .58 | .55 |
| X. Integumentive Organs, | 13 | 132 | .07 | .09 |
| XI. Old Age, | 997 | 8,481 | 5.10 | 6.12 |
| XII. Violent Causes, | 816 | 5,356 | 4.17 | 3.86 |

"By this it will be noticed that the class of diseases designated zymotics, and the diseases connected with the respiratory organs, have been most fatal during the year under consideration, 57.40 per cent. of all the deaths whose causes have been reported having been caused by them. This has been the case, also, in the twelve years and eight months in which registration has been carried on in Massachusetts."

Elaborate and special tables exhibit the ravages of cholera infantum, croup, dysentery, erysipelas, measles, typhus fever, scarlatina, scrofula, consumption, pneumonia, teething, and suicide.

We reduce the following from the several tables and accompanying comments:

| ZYMOTICS. | No. of Deaths 1853. | SEX. Males. | Females. | Unknown. | Per centage of all causes. 1853. | Per cent. 12 yrs. 8 mos., ending December 31, 1853. | MOST FATAL PERIOD OF LIFE. |
|---|---|---|---|---|---|---|---|
| Cholera, | 90 | 54 | 36 | | .46 | 1.13 | Between 30 and 40 yrs. |
| Cholera Infantum, | 535 | 265 | 267 | 3 | 2.74 | 2.03 | All but 5 under 5 yrs. |
| Croup, | 608 | 311 | 297 | | 3.11 | 2.28 | 495 u. 5 yrs., 103 b. 5 & 10. |
| Diarrhœa, | 243 | 122 | 121 | | 1.24 | 1.03 | 191 under 5 years. |
| Dysentery, | 1,046 | 516 | 518 | 12 | 5.35 | 7.54 | 705 under 5 years. |
| Erysipelas, | 175 | 93 | 82 | | .89 | .93 | 67 under 20 years. |
| Fever, | 399 | 207 | 188 | 4 | 2.04 | 1.97 | |
| " Intermittent, | 4 | | | | .02 | .02 | |
| " Remittent, | 1 | | | | .01 | .02 | |
| " Typhus, | 771 | 412 | 359 | | 3.94 | 5.04 | 334 bet. 15 and 30 yrs. |
| Hooping Cough, | 221 | | | | 1.13 | 1.02 | All but 4 under 5 yrs. |
| Influenza, | 39 | 13 | 26 | | .20 | .31 | 28 bet. 70 and 90 yrs. |
| Measles, | 226 | 100 | 126 | | 1.16 | 1.05 | The first five years. |
| Scarlatina, | 1,005 | 495 | 505 | 5 | 5.14 | 4.24 | 945 u. 10., 712 u. 5 yrs. |
| Small-pox, | 38 | | | | .19 | .49 | |
| Syphilis, | 7 | | | | .04 | .02 | |
| Thrush, | 38 | | | | .19 | .06 | 37 under 5 years. |
| | 5,446 | | | | 27.84 | 29.17 | |

There had been an increase in the number of deaths in the foregoing diseases over 1852, as follows:

Cholera increase, 30; cholera infantum increase, 158; croup, 179; diarrhœa, 111; dysentery, 28; erysipelas, 12; fever, 43; typhus, 106; hooping cough, 55; measles, 85; scarlatina, 162; small-pox, 5; syphilis, 4; thrush, 11. In influenza there had been a decrease of 29 deaths.

From an examination of the foregoing tables, it will be seen that the various grades of fever, dysentery, and scarlatina, were the three most fatal diseases of the year, belonging to this class.

In reference to diseases of the other classes we extract the following observations:

"*Diseases of the Nervous Organs.* From year to year, until the present, there has been a gradual increase of deaths by diseases of this class. The tables of this year's report show a diminution of six deaths. The diseases comprehended in this class are among the most important in the whole list of disorders to which mankind is subject. The whole number of deaths attributed to them has been, during the year 1853, 2,008, or 10.27 per cent. of all the deaths from specified causes; in the last five years 9,337; and 15,435 or 9.76 per cent. during the twelve years and eight months of Massachusetts registration. Hydrocephalus proved most fatal, (1853,) carrying off 446 victims; next followed diseases of the organs not particularized; and then epilepsy, cephalitis, paralysis, and convulsions."

"*Diseases of the Respiratory Organs.* This class of diseases has proved most fatal during the past year. The whole number of deaths amounted to 5,783, or 29.56 per cent. of all the diseases from specified causes.

The very large number of 25,350 persons died of diseases belonging to this class during the last five years. Consumption and pneumonia, the most interesting as well as the most prominent of its maladies, produced the last year 5,477 deaths out of 5,783, and will have a more extended notice.

"Of the 4,593 deaths caused by *consumption*, during the year 1853, 1,884 were of males, 2,707 of females, and of 2 the sex was not reported. The per centage compared with the deaths resulting from all other known causes, amounted to 23.48—an increase over that of previous years. The very large number of deaths of children under five years of age,—namely, 241 of males, and 185 of females—would seem to indicate that many of the complaints which usually prove fatal to young children had been incorrectly returned, and their results, consequently, added erroneously to the account of consumption. Unquestionably, on the other hand, many deaths justly attributable to this destructive malady have in their turn been set down to the various causes operating against infant life, as marasmus, cholera infantum, infantile diseases, &c.

"There were 1,226 fatal cases of consumption which occurred between the ages of 20 and 30—768 of males, and 458 of females. The next most fatal period was between the ages of 30 and 40, when 759 individuals died—293 males, and 466 females. Even as late as the period between the ages of 70 and 80, 222 deaths occurred.

"There is a remarkable evenness as to the time of the year in which the mortality happened. August and April of the year for which this report is prepared, proved the most fatal, and November and February the least so.

"In the course of the last five years this disease is reported to have proved fatal to 19,863: 3,606, or 18.37 per cent. in 1849; 3,527, or 21.96 per cent.

in 1850; 3,982, or 21.73 per cent. in 1851; and 4,155, or 23 per cent. in 1852. In the twelve years and eight months of registration there were 35,479 deaths, being 22.44 per cent. of all the deaths from specified causes. During the last five years, very nearly eleven individuals have died daily of this disease, and about thirteen a day during the year 1853."

"*Pneumonia.* This disease proves, next to consumption, the most fatal of the diseases of the respiratory organs. There were 884 fatal cases during the year, being 4.52 per cent. of all the deaths from known causes. Of these 476 were males, 402 females, and 6 of unknown sex. Among children under five years of age, the disease proved very fatal, 464 children of that period having died during the year. After this age, the middle period of life seems, from the tables, to have been most inauspicious in reference to this malady. Although March and April were the months in which the greatest mortality prevailed, yet there was a very great evenness in the occurrence of the deaths by this disease throughout the year."

Respecting the other diseases of this class there is nothing especially noteworthy.

"*Diseases of the Organs of Circulation.* The whole number of deaths under this class, in 1853, was 475, a decrease of five from previous year. Of these, two were attributed to *pericarditis*, and the remainder to diseases of the organs. During the five years ending Dec. 31, 1853, 2,161 persons died of diseases of this class, and for the whole period of registration 3,358 individuals are set down in the tables."

*Diseases of the Digestive Organs.* Fifteen diseases are embraced under this head, numbering 1,191 deaths. This is a decrease of 112 from the previous year.

Dentition is charged with 385 of the deaths, and enteritis with 235. Liver diseases are numerous, and are thus entered: Hepatitis, 5; jaundice, 17; diseases of the liver, 140. And 171 are put down to "ulceration." The balance of the figures is distributed to the remaining diseases of the classification.

*Diseases of the Urinary Organs.* The figures of the diseases in this class are as follows:

| | | | |
|---|---|---|---|
| Diabetes, - - - - - | 20 | Males, 10 | Females, 10 |
| Gravel, - - - - - - - | 23 | " 21 | " 2 |

| | | | | | | |
|---|---|---|---|---|---|---|
| Nephritis, - - - - - - | 3 | Males, | 3 | Females, | 0 |
| Cystitis, - - - - - - - | 6 | " | 4 | " | 2 |
| Prostatitis, - - - - - | 2 | " | 2 | " | 0 |
| Stranguary, - - - - - | 1 | " | 1 | " | 0 |
| Diseases Kidney, - - | 33 | " | 22 | " | 11 |

Total, 38 deaths.

*Diseases of the Generative Organs.* The deaths under this head are thus classed:

Child-birth, 197; puerperal fever, 21; diseases of organs, 4. Total 222. The number of deaths from puerperal fever in 1852, was 50; and in 1851, was 54.

To the *Diseases of the Locomotive Organs* are charged 113 deaths; and to the next class in the order, *Integumentive Organs*, 13 deaths.

*Old Age* is reported as the cause of 997 deaths, 391 being males and 606 females. This gives a little over 5 per cent. of those who died from old age. Of these 698 died over 80 years of age.

*Violence.* 82 persons were burned or scalded; 182 drowned; 8 frozen; 2 died of hydrophobia, and 42 of intemperance. The death of one person was acknowledged as by mal-practice; 11 persons were starved; 11 murdered; 16 poisoned; 12 suffocated; and 321 from accidents not enumerated.

Sixty-seven persons died in consequence of *suicide*—a decrease of 9 from the year 1852. Of these 47 were males and 20 females.

In illustration of the influence of occupation, or labor, upon the duration of life, lengthy tables are given of the *Occupations of the Deceased*, for which we have not room. The following deduction from the tables by the reporter, struck us as rather a novel fact:

"During the period of nine years and eight months, *paupers have, as a class*, died at the most advanced age considered in the aggregate. Then in succession, may be found agriculturists, public men, professional men, merchants, mechanics, laborers, and seamen."

This brings us in order to the close of the volume. Although conscious of not having done full justice to the Report, we have presented as large a share of its contents as our limit will permit. Sufficient has been given to afford good proof of the value of such statistics gathered throughout a state and by a parity of reasoning of their still greater value if they could be made to extend so as to inclose the entire Union.

The Registration Laws of our own State have proved inoperative from some defect in their mode of working. It is much to be regretted that a sufficient interest in the subject could not be awakened, so that existing defects could be remedied, and our quota furnished to the vital and mortuary statistics of the country. J. M. N.

March, 1855.

